# Community-based abdominal ultrasound survey of cystic echinococcosis in Bhutan: prevalence and risk factors

**DOI:** 10.1186/s12889-026-28325-w

**Published:** 2026-06-30

**Authors:** Pema Wangchuk, Jan Hattendorf, Jakob Zinsstag

**Affiliations:** 1 Swiss Tropical and Public Health Institute, Department of Epidemiology and Public Health, Kreuzstrasse 2, Allschwil, 4123 Switzerland; 2 University of Basel, Peterplatz 1, Basel, 4001 Switzerland; 3 Gidakom Hospital, Thimphu, 11001 Bhutan

**Keywords:** Cystic echinococcosis, Ultrasound screening, Zoonosis, One Health, Prevalence, Risk factors

## Abstract

**Background:**

Cystic echinococcosis (CE) is a neglected parasitic zoonosis, caused by larvae of the *Echinococcus granulosus sensu lato* (s.l.) complex. The parasitic transmission cycle is maintained between dog and livestock (ungulates), with humans involved as dead-end hosts. In Bhutan, CE is a recognized public health and veterinary problem; however, the true burden of CE in high-risk communities remains unknown. This study assessed the community-level prevalence, potential risk factors, and awareness of CE in Bumthang District to inform CE control strategies.

**Methods:**

We conducted a cross-sectional, community-based abdominal ultrasound survey (April–July 2025) in four gewogs of Bumthang district using cluster sampling design. We invited all individuals aged ≥ 12 years from the selected villages. The WHO-IWGE ultrasound staging criteria were used to classify the cysts.

**Results:**

Among the 3,058 participants with complete data (of 3,077 screened), the cluster-adjusted prevalence of confirmed CE (ultrasound-pathognomonic findings) was 2.58% (95% CI: 1.93–3.44). When cystic lesions (CL) were included, the estimate was 3.66% (95% CI: 2.92–4.59). The liver was the most commonly affected organ (93%), and cyst stages ranged from CE1 to CE5, indicating the presence of both active and inactive infections. Only intermittent dog contact (“sometimes”) was independently associated with higher odds of prevalent CE (aOR 1.73, 95% CI 1.04-2.82). Other commonly hypothesized individual-level factors (sex, education, occupation and place of birth) were not independently associated with infection. All participants with CE and other clinically significant ultrasound findings received counselling, stage-appropriate management for CE cases, and appropriate referral and treatment within Bhutan’s state-funded free healthcare system.

**Conclusion:**

CE is prevalent in Bumthang district. Currently, it remains under-recognized, under-reported and a huge detection gap exists between hospital-based passive detection and true community prevalence. The weak discrimination by individual-level risk factors and the pattern of modest village-level clustering are consistent with the environmentally mediated transmission hypothesis. These findings support the need of One Health control strategies including integrated surveillance rather than targeted approaches for high-risk groups.

**Supplementary Information:**

The online version contains supplementary material available at https://doi.org/10.1186/s12889-026-28325-w.

## Introduction

### Background

Cystic echinococcosis (CE) is caused by the larval stage of the *Echinococcus granulosus sensu lato* (s.l.) complex. CE remains a globally significant zoonotic disease that affects both human and animal health, particularly pastoral and livestock rearing communities [1]. Despite sustained control efforts, CE continues to pose a major public health and economic burden in many endemic regions, including Central Asia, South America, parts of Africa, and the Himalayas [2]. The number of global incident CE cases is projected to rise from approximately 207,000 in 2019 to approximately 236,000 by 2030, an increase of 13.7% over this period [3]. The World Health Organization (WHO) recognizes CE as a neglected tropical disease (NTD) and a high-burden foodborne parasitic disease, with a high disability attributable to its chronic nature and the need for surgical intervention for active disease [4, 5].

CE is closely associated with livestock rearing, the presence of free-roaming dogs, and poor hygiene and sanitation practices [6]. In pastoral communities where dogs have close contact with livestock, humans are primarily exposed to *Echinococcus *spp. infections through contaminated food and water [7]. A recent One Health study from South Asia highlighted that low awareness, poor hygiene, and informal slaughtering practices significantly increase the risk of exposure [8]. CE is typically asymptomatic for years while cysts grow slowly in affected organ(s). As cysts enlarge, they can cause abdominal pain, biliary obstruction, cholangitis, and, if a cyst ruptures, anaphylaxis or secondary dissemination. Untreated hepatic CE carries appreciable morbidity, whereas early detection and timely stage-specific management (watch-and-wait for inactive cysts, albendazole, PAIR, or surgery) substantially reduces complications [9, 10].

Bhutan’s agropastoral and socio-cultural context is characterized by scattered and sparse rural settlements, free-ranging livestock grazing, and close dog-human interactions [11–13], factors that facilitate parasite persistence and transmission. A recent retrospective hospital-based study documented the burden and spatial distribution of CE in Bhutan, indicating the need for improved disease surveillance and control measures [14]. Molecular analyses conducted by the National Center for Animal Health have confirmed the presence of *E. granulosus sensu stricto *(s.s.) (G1-3 genotypes) and *E. ortleppi* (G5 genotype) in cattle, a dominant livestock species in the country, and dogs, indicating active transmission cycles among domestic hosts [15, 16]. In Bhutan, only G1 and G3 genotypes have been identified in human CE cases to date. Although human infection due to the *E. ortleppi* genotype is rare, the emergence of human cases in Asia, including South Asia, has been reported [17]. A hospital-based study that reported cases from multiple districts across the country and molecular confirmation of the parasite’s presence in the environment indicates silent community transmission. Most cases remain underdiagnosed because of the lack of disease surveillance and diagnostic infrastructure, particularly in rural areas [14, 18].

To date, epidemiological data on CE in Bhutan have been mainly derived from hospital-based records and isolated case reports [14, 19–21]. No population-based ultrasound surveys have quantified the prevalence of CE or evaluated exposure patterns in the country. Understanding the epidemiology of CE in Bhutanese communities is critical for developing effective One Health control strategies, including the integration of CE surveillance into broader ‘priority-zoonotic disease’ surveillance and control programs. This study aimed to determine the community prevalence of CE and its associated risk factors in a high-risk district, fill existing knowledge gaps, and contribute evidence towards informing national CE control strategies.

## Materials and methods

### Study design and setting

**Fig. 1:**
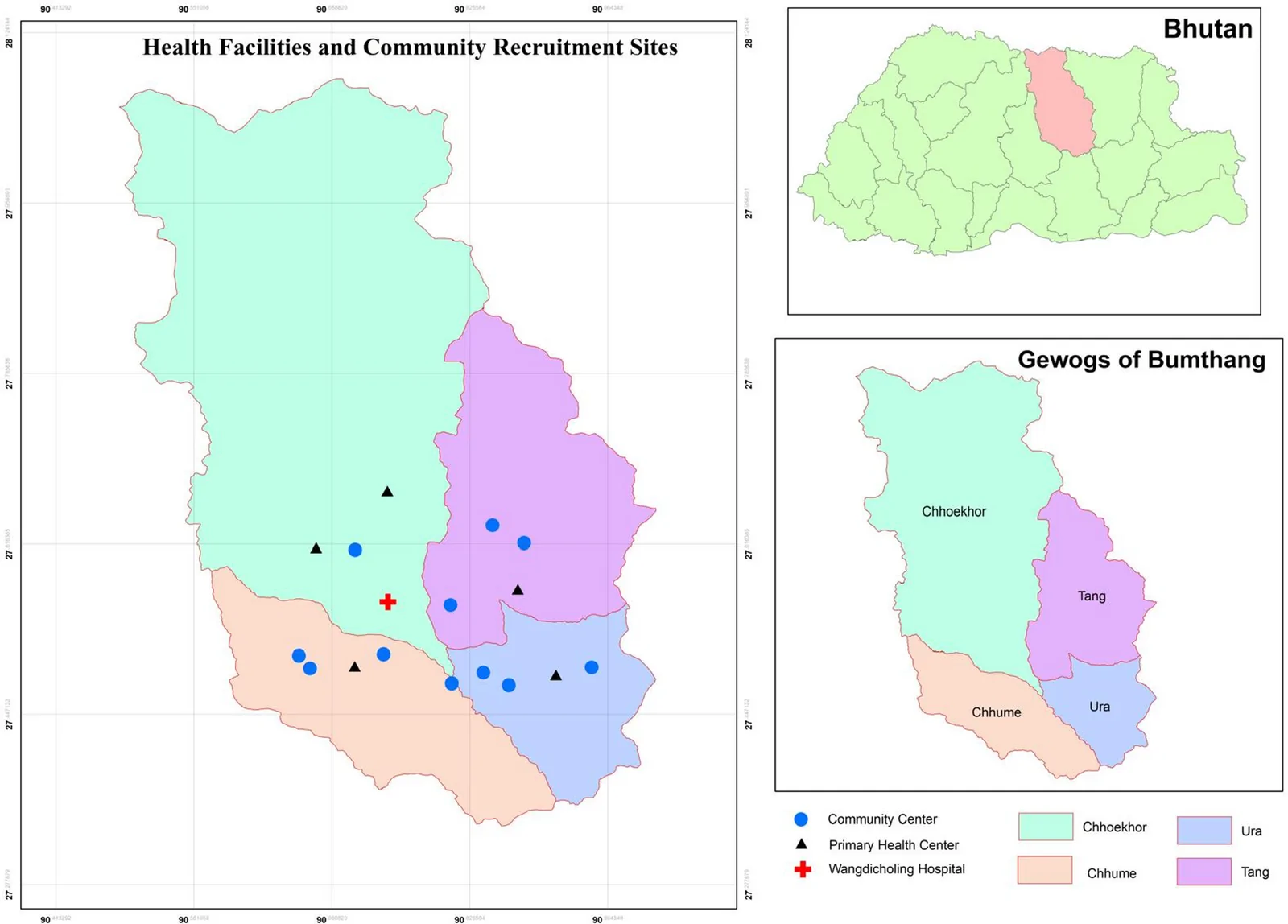
Study site showing the district hospital, PHCs and community centers for participants recruitment

This cross-sectional, community-based study was conducted between April and July 2025 in Bumthang district. As shown in Fig. 1, Bumthang district is in the North-central region, situated at approximately 27.5°–27.9° N and 90.6°–91.1° E latitudes. The district has four major river valleys, characterized by high-altitude Himalayan ecology, with elevations ranging between 2,600 and 4,500 m above sea level. The majority of residents’ livelihoods are largely based on subsistence agriculture, livestock farming, and seasonal pastoralism, particularly yak and cattle herding at higher elevations during warmer summer months. Bumthang has a predominantly rural population of approximately 17,000 residents, with scattered settlements and a relatively low population density [22].

Bhutan has a three-tiered, state-funded health system in which the state provides free healthcare services to its citizen, including the full cost of ex-country referral for treatment that is not available in the country [23]. In line with the country’s health service delivery, health services in Bumthang are delivered through one district general hospital (Wangdicholing Hospital), five primary health centers (PHCs), and outreach clinics that serve remote communities across four gewogs (administrative blocks). Access to diagnostic imaging (ultrasonography) services is limited outside the district hospital catchment area, particularly in rural and semi-nomadic areas, making Bumthang a relevant setting for community-based ultrasound screening for CE.

The study sites comprised rural areas of four gewogs, selected as pilot sites due to previously reported higher CE burden/cases from hospital reports. Chamkhar town and villages within the catchment area of the district hospital were excluded from the sampling frame because residents in these areas had routine access to diagnostic ultrasound services through the district hospital. The survey was designed to assess the burden of previously undiagnosed cystic echinococcosis in communities with limited access to ultrasound screening. Participants were identified from PHCs registries and recruited in collaboration with local (*chiwog* and village-heads) leaders, including a limited number of semi-nomadic pastoral households engaged in yak herding in the district’s northern region.

### Study population, sample size and sampling

The target population included permanent residents of selected *chiwogs* and villages in the Bumthang district, including semi-nomadic pastoralist communities. Eligible participants were individuals aged ≥ 12 years who had resided in the selected villages for at least six months, provided informed consent (or assent for minors), and were present during the survey. Individuals with acute or severe illnesses that prevented attendance and temporary residents were excluded.

The sample size was calculated to estimate the CE prevalence with a precision of 2% points (half-width of the 95% CI), assuming a prevalence of 4% and an intraclass correlation coefficient (ICC) of 0.05. With an average cluster size of 30 individuals, the corresponding design effect was estimated to be 2.45. We estimated that we required a minimum of 38 clusters and 1,140 individuals and inflated this number by 10% to account for non-response [24]. Sampling followed a cluster sampling design using probability proportional to size (PPS). The sampling frame comprised all villages in Bumthang district outside of Chamkhar town and the district hospital catchment area. Villages formed primary sampling units (PSU), and forty-four villages were selected by PPS based on the number of households. Within each selected village, all households were enumerated and all eligible members were invited to participate in ultrasound screening and questionnaire interviews. This complete enumeration minimized within-village selection bias and enabled inclusion of residents across a broad range of demographic and socioeconomic characteristics. A total of 3,077 individuals from 44 villages participated in the survey.

### Data collection procedures & quality assurance

Ultrasound examinations and questionnaire interviews were conducted at PHCs or community centers based on participants accessibility. Abdominal ultrasound examinations were performed using Mindray M6 and MX7 machines with curvilinear transducers in the conventional B-mode, following a standardized scanning protocol. Abdominal ultrasound is the recommended first-line diagnostic tool for hepatic CE, with high reported diagnostic performance in experienced hands, particularly for active cyst stages (CE1-CE3), based on comparative imaging studies and expert consensus [9, 25, 26]. Examinations were performed by the principal investigator and an experienced (> 10 years) medical sonographer from the Wangdicholing District Hospital. The examinations were performed once by each examiner. In cases of uncertainty, exams were repeated by the other and findings discussed to reach consensus. Ultrasound examinations were performed independently of the questionnaire; sonographers were not blinded to participant identify but did not review questionnaire responses before scanning. Data on sociodemographic characteristics, dog-related practices, food handling and hygiene behaviors, environmental exposures, and awareness of CE were collected using a structured questionnaire developed specifically for this study (Supplementary Table S3). The questionnaire was pilot tested in 2 non-study villages and items were revised accordingly for clarity, length and appropriateness prior to the main survey. Participants completed the questionnaire before the ultrasound examination.

As part of the data quality assurance, sonographer and data collectors/field staff were trained on the study protocol, and briefings were provided before each session. Each examination was performed once by one of the sonographers; a subset of ultrasound images of CE cysts was independently double-read to assess inter-observer agreement. Field supervisors and the principal investigator checked the completeness and consistency of the questionnaires daily on Open Data Kit (ODK) Collect. ODK (https://getodk.org) is an open-source mobile data collection platform that supports online and offline data collection in the field. After verification, complete questionnaire forms were submitted to ODK Central. Data validation rules were applied during data entry in the ODK collect forms.

### Data management and statistical analysis

Data were downloaded from ODK Central as comma-separated value files. The datasets were checked for completeness and internal consistency. Range and logic checks were applied to identify outliers and implausible values in the data. Duplicate records were removed, variables were re-coded as required, and the distributions of continuous variables (age and cyst size) were examined and appropriately described. All analyses were conducted using R software (version 4.4.3).

### Case definition and study variables

Confirmed CE cases were defined as ‘individuals with ultrasound findings pathognomonic of echinococcal cysts’, and suspected cases were defined as ‘individuals with ultrasound findings consistent with, but not pathognomonic of echinococcal cysts.’ To avoid systematic underestimation, suspected cases included CL, which are considered ‘potentially echinococcal in the absence of advanced imaging or serology’ [9, 27]. Both confirmed and suspected cases were combined into CE cases, and a single binary outcome (CE vs. non-CE) was used in all analyses. Socio-demographic variables included sex and age, which was categorized as < 19, 20–39, 40–59, and ≥ 60 years. Other categorical variables included education, occupation, and place of birth (Bumthang natives versus other districts). Behavioral exposures included frequency of dog contact (always/often/sometimes/rarely/never) as a categorical exposure variable, raw meat consumption (yes/no), and handwashing (always/often/sometimes/rarely/never).

Among the CE cases, the cyst stage was classified according to the World Health Organization-Informal Working Group on Echinococcosis (WHO-IWGE) ultrasound classification. The cyst stages and cyst activity were analyzed and described at the cyst level. Person-level summaries were derived for the number of cysts, cyst location, and cyst diameter.

### Statistical analysis: risk factor modelling and model selection

#### Prevalence estimation

Prevalence analyses were based on all screened individuals with complete ultrasound results and village identifiers (*n* = 3,058). The prevalence of confirmed, suspected, and combined CE (confirmed and suspected) was calculated as the proportion of cases among those screened with complete data. To account for the clustered sampling design, we used intercept-only logistic regression models with cluster-robust standard errors (sandwich estimator) and clustering adjustment at the village level. This approach avoids the instability of mixed-effects models for rare outcomes while properly adjusting for within-village correlation. The point prevalence was obtained by back-transforming the model intercept to the probability scale using the inverse logit function as follows:

Prevalence = exp(β₀) / (1 + exp(β₀)).

Cluster‑robust 95% confidence intervals were calculated on the log-odds scale and then back‑transformed to the probability scale. Prevalence and subgroup-specific estimates were calculated using confirmed CE cases in cluster-robust model.

### Risk factor analysis

For risk factor analyses, we used a combined outcome that included confirmed CE and suspected cases, because CL lesions are considered potentially echinococcal under the WHO-IWGE classification and their inclusion improved model stability. Confirmed-only models were explored as a sensitivity analysis where estimable. Odds ratio presented in Tables 3 and 4, and the ICC estimates were derived from the mixed-effects model.

#### Univariable analysis

Univariable and risk factor analyses were restricted to participants with complete covariate data (*n* = 3,058). The associations between CE and each explanatory variable were assessed individually using mixed-effects logistic regression models with a village-level random intercept. Crude odds ratios and 95% profile-likelihood confidence intervals (for better small-sample coverage with sparse cells) were estimated using these models. For each variable, a global p-value was obtained from a likelihood ratio test (LRT) comparing the model with the predictor to the intercept-only model using the drop1() function. Variables with a global LRT p-value < 0.25 were considered candidates for the multivariable model to avoid excluding potentially important confounders. Age group and sex were retained a priori, regardless of their univariable p-values.

#### Multivariable modelling

A multivariable mixed-effects logistic regression model with a village-level random intercept was fitted to estimate adjusted odds ratios (aORs) and 95% confidence intervals. The model performance was assessed using convergence diagnostics and singularity checks (no singular fit was detected). The intraclass correlation coefficient (ICC) was calculated from the village-level random-effects variance as σ²_u_ / (σ²_u_ + π²/3). As a sensitivity analysis, alternative clustering structures were explored, including household-level clustering and households nested within the villages. These models were compared using the Akaike Information Criterion (AIC), Bayesian Information Criterion (BIC), and likelihood ratio tests. The village-only random intercept model was retained as the primary analysis model because household-level clustering was statistically unstable due to sparse binary outcomes and a median household size of two. All analyses were conducted using R software (version 4.4.3), with mixed-effects models fitted using the *lme4 package*, model diagnostics assessed using the performance package, and tables prepared using the *gtsummary* package.

This study was reported in accordance with the Strengthening the Reporting of Observational Studies in Epidemiology (STROBE) guidelines for cross-sectional studies [28] (Supplementary Table S4).

### Ethical considerations

All study procedures during the survey were conducted in accordance with the Declaration of Helsinki. The study protocol, which included a prespecified ancillary care plan within the context of Bhutan’s free health system, was reviewed and approved by the Research Ethics Board for Health, Ministry of Health, Bhutan (approval number: *Ref. No./PO/RL/2024.1.NNW*). In addition, the study protocol was reviewed by the Ethikkommission Nordwest-und Zentralschweiz, Switzerland (Study ID: *AO_2024_00045*). Informed consent was obtained from all adult participants, and informed parental or legal guardians consent and informed assent for minors (< 18 years) after explaining the study objectives, procedures, risks, benefits and voluntary participation orally in Dzongkha or the local dialect (*Bumthap)*. For participants who were not able to read, the information sheet (Dzongkha version) was read aloud in the presence of a witness, and informed consent was documented by a thumb impression. Participants with clinically significant ultrasound findings, including CE, were treated, counselled on their diagnoses and referred to the nearest health facility for further evaluation and management. Similarly, participants with significant non-CE findings received on-site counselling and referral to the nearest health center as appropriate. Individuals with sonographic fatty liver disease were provided lifestyle counselling aligned with the WHO Package of Essential Noncommunicable Disease interventions (WHO PEN) [29]. All required additional diagnostic work-up and treatment were provided within the country’s state-funded, free healthcare system, at no cost to the patients. All data were pseudo-anonymized and handled confidentially.

## Results

### Study population characteristics

Of the 3,077 screened participants, 3,058 with complete data were included in the prevalence and risk factor analyses. Participants were from 1266 distinct households across 44 villages from four gewogs of Bumthang district. The median household size was 2 (IQR: 1–3). Sex and age distribution of the study population are presented in the Table 1. The study population had a median age of 43 years (IQR: 30–59 years).

As presented in Tables 1 and 82% of the study participants were natives, and the remaining (18%) migrated from other districts. Among non native participants in Bumthang, 73% (*n* = 376) had lived in the district for more than five years, 15% (*n* = 79) for one to five years, and 12% (*n* = 62) for less than one year. The occupational and educational characteristics of the study participants are presented in Supplementary Table S1.

**Table 1:** Baseline demographic characteristics of the study participants

Characteristic	N	n (%)
Sex	3,058	
Male		1,238 (40%)
Female		1,820 (60%)
Age Group	3,058	
<19		411 (13%)
20-39		937 (31%)
40-59		986 (32%)
60+		724 (24%)
Place of Birth	2,842	
Native		2,325 (82%)
Non-native		517 (18%)

### Prevalence of CE

Among the 3058 screened individuals, 112 were CE-positive (79 confirmed and 33 suspected). After adjusting for village-level clustering, the combined adjusted prevalence of CE was 3.66% (95% CI: 2.92–4.59), cluster-adjusted prevalence of confirmed CE was 2.58% (95% CI: 1.93–3.44), and cluster-adjusted prevalence of suspected CE was 1.08% (95% CI: 0.76–1.53). Of the 79 confirmed CE cases, 26.6% (21/79) had been previously diagnosed.

**Fig. 2:**
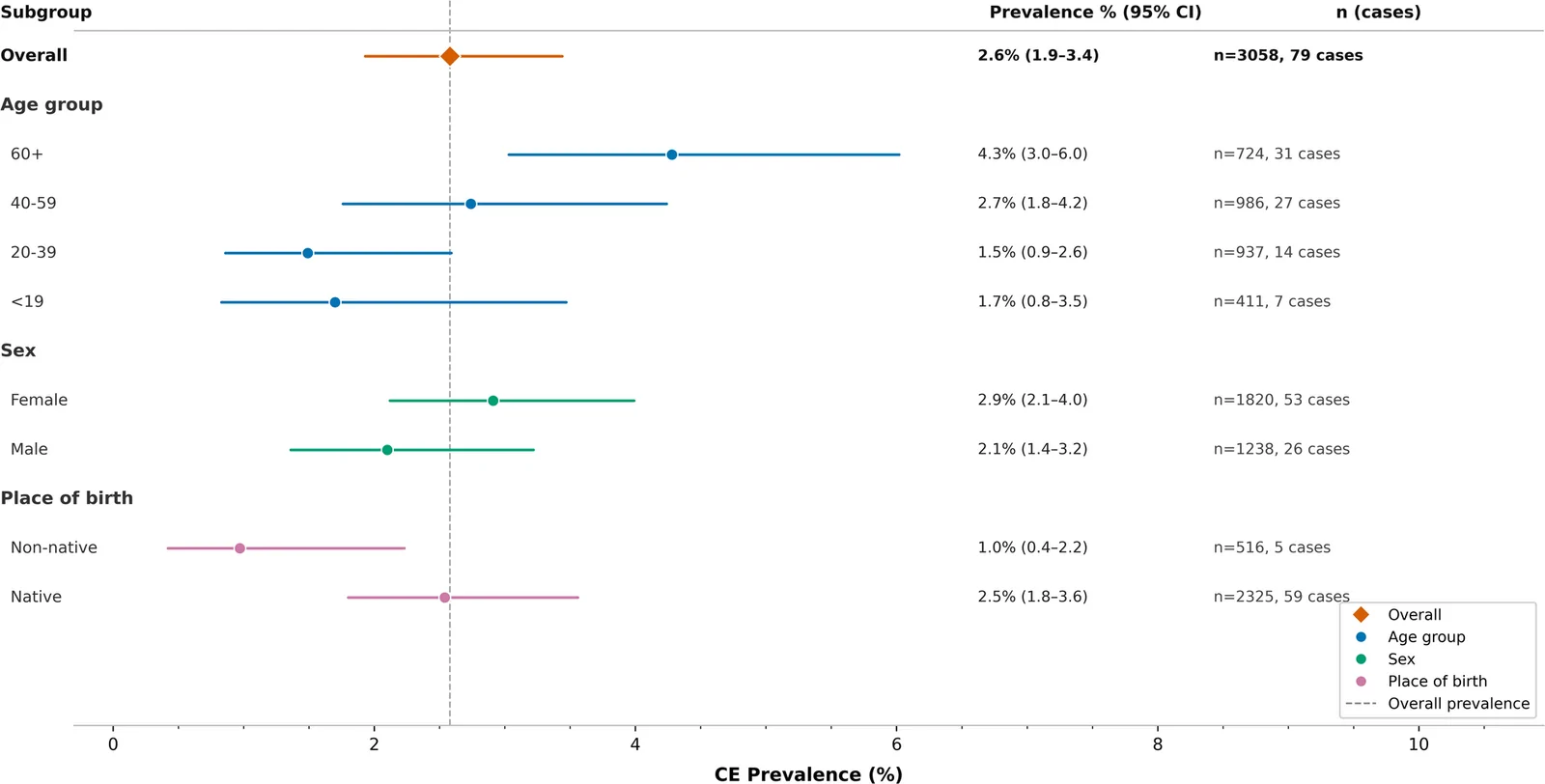
Forest plot of cluster-adjusted CE prevalence by age group, estimated by intercept-only logistic regression with cluster-robust standard errors

The confirmed CE prevalence and subgroup estimate for age-groups, sex and place of birth are shown in Fig. 2. Individuals with no formal education had the highest prevalence (4.4%), whereas a lower prevalence was observed among those with secondary or higher education. By occupation, farmers had the highest prevalence (4.3%), followed by students (1.7%) and other occupational groups.

### Incidental (non-CE) ultrasound findings

Incidental non-CE findings were detected in 796 participants (26%). In 26 cases, specific incidental findings were not recorded. Among 770 with recorded incidental findings, sonographic fatty liver disease accounted for 85.6% (*n* = 659) of these cases, followed by gallstone disease (cholelithiasis) at 9.9% (*n* = 76). Less frequent findings included simple renal cysts at 2.1% (*n* = 16), hepatic hemangioma at 0.8% (*n* = 6), renal stones at 0.4% (*n* = 3), hydronephrosis at 0.4% (*n* = 3), and ovarian cysts at 0.3% (*n* = 2). Among participants with fatty liver, Grade I steatosis was most common (59.0%, *n* = 389), followed by Grade II (28.4%, *n* = 187) and Grade III (0.9%, *n* = 6). In 11.7% (*n* = 77), grading was not recorded.

### Geographic distribution and awareness

**Fig. 3:**
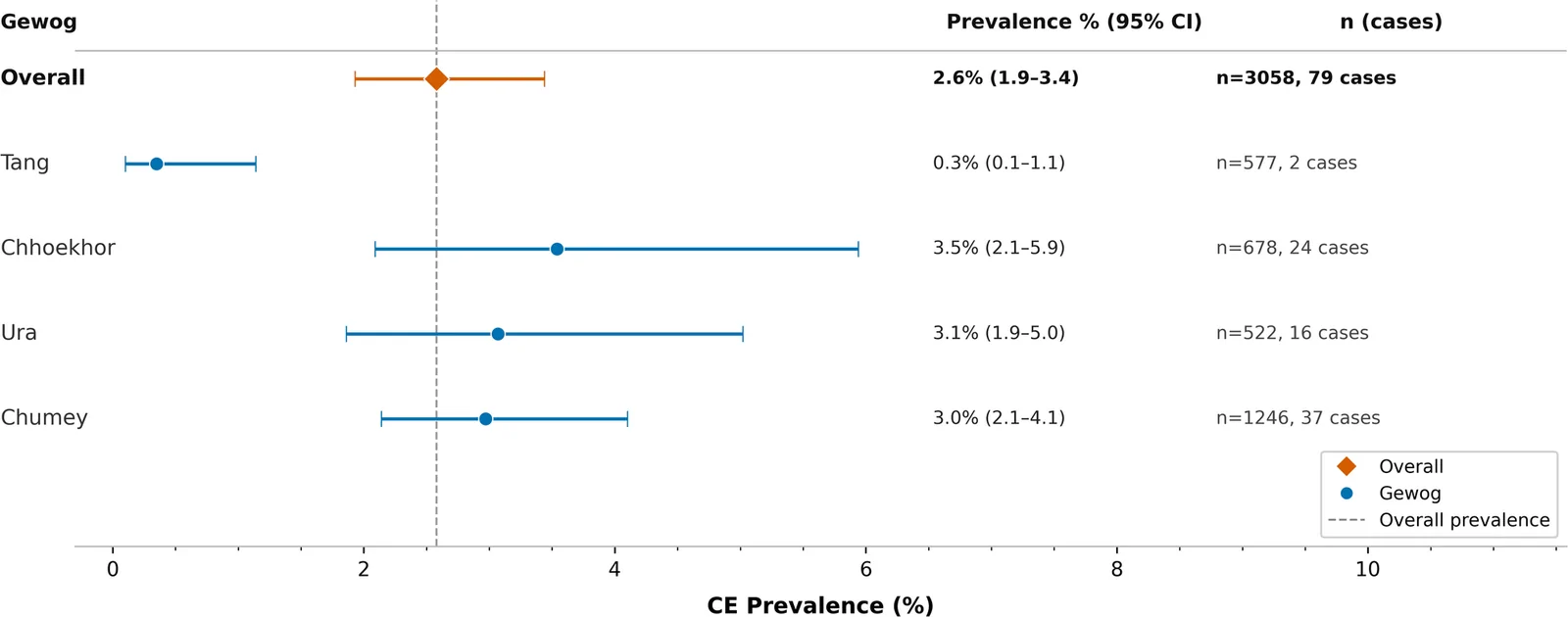
Forest plot of cluster-adjusted CE prevalence by gewog, estimated by intercept-only logistic regression with cluster-robust standard errors

As shown in Fig. 3, the cluster-adjusted gewog-wise CE prevalence (confirmed) varied across the four gewogs in the district, ranging from 0.3% in Tang gewog to 3.5% in Chhoekhor gewog. The highest number of CE cases was detected in Chumey (37/1246), with a cluster-adjusted prevalence of 3.0% (95% CI: 2.14–4.10), whereas the lowest number of cases was detected in Tang (2/57).

Awareness of CE and reporting of a family member affected by CE showed only modest differences between CE and non-CE cases (20.7% vs. 14.9%, and 4.3% vs. 1.3%, respectively). Overall, disease awareness of CE and family history of the disease were not frequently reported in the study population, with only 15.1% (428/2842) aware of CE and 1.4% (40/2842) reporting a family member affected by the disease.

### CE cyst characteristics

Among individuals with CE cysts, 114 cysts were recorded in 104 of the 112 individuals with documented cyst count data; nine had multiple cysts (≥ 2). Of 110 classified cysts, 56.4% were active (CE1–CE3B), 20.0% inactive (CE4–CE5), and 23.6% were CL lesions. The most frequent stages were CE1 (26.4%) and CL (23.6%). Most affected individuals had a single cyst (91.3%), the liver was the predominant site (92.9%), and the median cyst diameter was 5.9 cm (IQR: 4.0–8.2 cm). Full cyst characteristics are presented in Table 2.

**Table 2:** Cyst characteristics among individuals with CE

Characteristic		*n* (%)
WHO-IWGE ultrasound stage (cyst-level, *n* = 108)		
	CE1	27 (25.5)
	CE2	7 (6.6)
	CE3A	5 (4.7)
	CE3B	17 (16.0)
	CE4	9 (8.5)
	CE5	12 (11.3)
	CL	26 (24.5)
	Mixed stages	3 (2.8) ᵃ
Disease activity (cyst-level, *n* = 110)		
	Active (CE1–CE3B)	62 (56.4)
	Inactive (CE4–CE5)	22 (20.0)
	CL (indeterminate)	26 (23.6)
Disease activity (person-level, *n* = 106) ᵇ		
	Active (CE1–CE3B)	59 (55.7)
	Inactive (CE4–CE5)	21 (19.8)
	CL (indeterminate)	26 (24.5)
Cyst location (person-level, *n* = 112)		
	Liver	104 (92.9)
	Kidney	4 (3.6)
	Liver and kidney	1 (0.9)
	Liver and lung*	1 (0.9)
	Lung*	1 (0.9)
	Mesentery*	1 (0.9)
Number of cysts per person (*n* = 104) ᶜ		
	1	95 (91.3)
	2	8 (7.7)
	≥ 3	1 (1.0)
Largest cyst diameter (*n* = 106) ᵈ		
	< 5 cm	39 (36.8)
	5–9.9 cm	54 (50.9)
	≥ 10 cm	13 (12.3)

ᵃ Mixed stages: CE1 & CE3B (n=1), CE2/CE1/CE3B (n=1), CE3B & CE4 (n=1); classified as Active where any active stage present

ᵇ 6 persons had no ultrasound classification recorded and are excluded from this denominator

ᶜ 8 persons had missing cyst number data; percentages based on n=104 with recorded data

ᵈ 6 persons had missing cyst size data; percentages based on n=106. *From previously diagnosed cases

### Risk factor analysis

The crude and adjusted associations, with per-category likelihood ratio p-values, for sociodemographic and behavioral factors derived from mixed-effects logistic regression with a village-level random intercept are presented in Tables 3 and 4. Numbers (N) vary by variable due to missing data; multivariable models were fitted using complete-case data (*n* = 2,841). Handwashing and raw meat consumption were not associated with CE infection (Supplementary Table S2).

**Table 3:** Univariable analysis of factors associated with CE, estimated by mixed-effects logistic regression with a village-level random intercept

Characteristic	*N*	*n* CE (%)	Crude OR (95% CI)	*p*-value
Sex				
Male	1,238	36 (2.9)	1 (Ref.)	
Female	1,820	76 (4.2)	1.45 (0.97–2.19)	0.07
Age group (years)				
< 19	411	7 (1.7)	1 (Ref.)	
20–39	937	16 (1.7)	0.95 (0.40–2.49)	0.90
40–59	986	38 (3.9)	2.21 (1.04–5.48)	0.04
60+	724	51 (7.0)	4.28 (2.04–10.47)	< 0.001
Education¹				
No formal education^*^	1,259	55 (4.4)	1 (Ref.)	
Non-formal education^**^	119	5 (4.2)	1.04 (0.35–2.46)	0.94
Primary level	407	14 (3.4)	0.76 (0.40–1.34)	0.36
Secondary/High school	746	8 (1.1)	0.23 (0.10–0.47)	< 0.001
Diploma/College and above	154	5 (3.2)	0.71 (0.24–1.64)	0.45
Monastic education	157	5 (3.2)	0.71 (0.24–1.65)	0.45
Occupation				
Farmer	2,139	91 (4.3)	1 (Ref.)	
Student	423	7 (1.7)	0.39 (0.16–0.79)	0.01
Businessperson	106	3 (2.8)	0.65 (0.16–1.79)	0.45
Civil servant	100	3 (3.0)	0.69 (0.17–1.89)	0.51
Monk/nun/monastic/military	70	1 (1.4)	0.33 (0.02–1.52)	0.19
Others	220	7 (3.2)	0.72 (0.30–1.48)	0.39
Place of birth				
Native of Bumthang	2,325	83 (3.6)	1 (Ref.)	
From other districts	516	9 (1.7)	0.48 (0.22–0.91)	0.02
Dog contact frequency				
Never	1,813	60 (3.3)	1 (Ref.)	
Rarely	168	3 (1.8)	0.56 (0.13–1.53)	0.29
Sometimes	651	27 (4.1)	1.31 (0.80–2.08)	0.27
Often	114	1 (0.9)	0.27 (0.02–1.24)	0.11
Always	96	1 (1.0)	0.32 (0.02–1.48)	0.18

Crude ORs from mixed-effects logistic regression with village-level random intercept. ¹Education data available for 2,842 participants (216 missing)

* No formal education refers to “individuals who have not attended formal schooling and are illiterate.” Non formal education** refers to “individuals who received structured functional literacy, numeracy, or life skills training, who missed the formal school system”

In the univariable analysis (Table 3), 40-59 and 60+ age groups, secondary/high school education, student, and non-native residency were the factors associated with CE. However, in the multivariable analysis (Table 4), only intermittent (“sometimes”) dog contact was statistically significant after adjusting for other variables. The association between intermittent dog contact (“sometimes”) and prevalent CE strengthened after multivariable adjustment: the crude OR (village random-intercept model) was 1.31 (95% CI: 0.80–2.08, *p* = 0.27), whereas the adjusted OR increased to 1.73 (95% CI: 1.04–2.82, *p* = 0.04), a 24% change in the odds ratio. This change is consistent with negative confounding by age, education, and occupation, which differ markedly between the “never” and “sometimes” contact groups: the “never” group was older (26.3% aged ≥ 60 vs. 14.7%), had less formal education (50.4% vs. 29.2%), and comprised more farmers (74.6% vs. 53.8%), all factors independently associated with higher CE prevalence. They increased the baseline risk in the reference group, which reduced the observed crude difference between groups.

**Table 4:** Multivariable analysis of factors associated with CE, estimated by mixed-effects logistic regression with a village-level random intercept

Characteristic	aOR (95% CI)	*p*-value
Sex		
Male	1 (Ref.)	
Female	1.20 (0.76–1.93)	0.45
Age group (years)		
< 19	1 (Ref.)	
20–39	1.34 (0.24–8.25)	0.75
40–59	2.10 (0.36–13.02)	0.43
60+	4.27 (0.75–27.0)	0.12
Education		
No formal education	1 (Ref.)	
Non-formal education	1.34 (0.44–3.29)	0.57
Primary level	1.29 (0.60–2.59)	0.50
Secondary/High school	0.42 (0.16–1.01)	0.06
Diploma/College and above	1.01 (0.25–3.42)	0.99
Monastic education	1.14 (0.34-3.04)	0.81
Occupation		
Farmer	1 (Ref.)	
Student	1.22 (0.21–6.00)	0.82
Businessperson	1.48 (0.34–4.37)	0.55
Civil servant	2.10 (0.42–7.71)	0.34
Monk/nun/monastic/military	0.40 (0.02–2.48)	0.37
Others	1.40 (0.54–3.10)	0.46
Place of birth		
Native of Bumthang	1 (Ref.)	
From other districts	0.51 (0.23–1.03)	0.06
Dog contact frequency		
Never	1 (Ref.)	
Rarely	0.59 (0.14–1.63)	0.35
Sometimes	1.73 (1.04–2.82)	0.04
Often	0.32 (0.02–1.50)	0.18
Always	0.38 (0.02–1.80)	0.27

Estimates from the mixed-effects logistic regression with village-level random intercept. Age and sex retained a priori

## Discussion

### Main findings

This community-based ultrasound survey, the first of its kind in Bhutan, screened 3,077 individuals aged ≥ 12 years across 44 villages in Bumthang district. Our prevalence (2.58%) falls within the range reported from comparable endemic settings: 0.7–4.9% across the Qinghai-Tibet Plateau and rural Mongolia [6, 30, 31]. Although, prevalence estimate cannot be directly compared with the reported hospital-based annual incidence of approximately 4.4 per 100,000 between 2014 and 2019 [14], the difference remains significantly large. This is consistent with the well-documented substantial under-reporting and detection gap between passive case detection and the true community burden in endemic regions [9, 18], and confirms that CE is a substantial yet under-recognized public health problem in Bumthang district.

In addition, our findings from the risk factor analysis are consistent with environmentally mediated transmission hypothesis in endemic settings, where exposure risks are widespread at the community level. The weak discrimination by individual-level factors is consistent with recent molecular and ecological evidence from South America, Iran, and China, which showed that *Echinococcus *spp. contamination levels can be similar in households with and without detected cases [32, 33]. The ICC of approximately 3.3% indicates that a small but meaningful fraction of CE variation is attributable to inter-village differences, suggesting exposure is broadly distributed across the district rather than confined to a few highly clustered villages, a pattern consistent with environmentally mediated transmission sustained by free-roaming dogs [34]. In such settings, interventions targeting only dog-owning households or specific occupational groups are unlikely to be sufficient; integrated One Health strategies, such as sustained dog deworming, vaccination of intermediate hosts, safe offal disposal, improved slaughter hygiene, and community-wide health education are required [18, 32].

### Cyst characteristics

Among the 112 individuals with CE, the liver was affected in 93.0% of the cases, consistent with the known organotropism of *E. granulosus* s.l. [9, 30]. The detection of active CE1 cysts indicates ongoing community transmission [27]. The case mix of active and inactive stages across age groups also indicates long-standing infections accumulated over years of environmental exposure, a pattern reported in similar community surveys from Uzbekistan, Chile, and Mongolia [6, 32, 35].

The proportion of active cysts is clinically important because these cyst stages harbor viable protoscoleces and typically require intervention based on the cyst stage, such as albendazole, percutaneous treatment, or surgery, depending on the cyst characteristics [9, 10, 36]. The co-existence of inactive cysts in approximately one-fifth of cases, likely representing spontaneous cyst involution, adds to the overall disease burden and requires ongoing surveillance [10, 35]. The fact that most cases were previously undiagnosed suggests the need for active community screening programs.

### Risk factors

The adjusted odds ratio for “sometimes” dog contact frequency category reached statistical significance (aOR 1.73, 95% CI: 1.04–2.82, *p* = 0.04), whereas all other categories showed no association. The strengthening from the non-significant crude OR of 1.31 (95% CI: 0.80–2.08) shows negative confounding. The “never” group was older, had lower education, and included more farmers than the “sometimes” group. These factors are independently associated with higher CE risk and increased baseline prevalence in the reference group, which likely reduced the apparent difference in the unadjusted analysis [37, 38]. The absolute prevalence differences across groups were small, and the isolated “sometimes” signal did not support a meaningful graded exposure effect. Self-reported dog-contact frequency is a coarse proxy for fecal-oral transmission risk in settings where free-roaming dogs are ubiquitous and environmental contamination is widespread [6, 7, 39]. This unusual pattern likely reflects residual confounding, an artefact of sparse outcomes in the exposure categories (sparse-data bias), false positive finding due to multiple comparison (without statistical correction), or also due to exposure misclassification rather than a distinct biological pathway and should be interpreted with caution.

The consistent upward gradient of prevalent CE with age is consistent with the chronic natural history of CE, in which prevalence reflects cumulative lifetime exposure in an endemic setting rather than recent infection events [26]. Population surveys in Tibet have documented a statistically significant positive age-prevalence gradient across more than 80,000 individuals [30], consistent with the biological plausibility of our findings.

Several commonly hypothesized risk factors, such as sex, occupation, and frequent dog contact, were not independently associated with prevalent CE after adjustment. Farmers constituted 70% of our study population, with a crude prevalence of 4.25%; however, occupation was not a significant predictor in the adjusted model. The absence of an occupational gradient, despite near-universal farming, is consistent with reports from mixed farming communities in Tibet where livestock rearing is too common to discriminate risk [30].

Similarly, individuals with no formal education had a crude prevalence of 4.37% compared with 1.07% among those with secondary or higher education. Education was not significant in the adjusted model. However, the overall protective effect of education, consistent with findings from community surveys in Mongolia and Yunnan province, China [6, 40], where education appeared to act as a structural factor shaping long-term hygiene and livestock-handling behavior rather than conferring immediate protection, can generally be inferred.

Although not significant in the adjusted model, individuals born outside Bumthang had lower adjusted odds of CE (aOR 0.51, 95% CI: 0.23–1.03, *p* = 0.06). This is consistent with the cumulative exposure effect, in which long-term residence in an endemic environment increases infection probability over time. Detailed migration histories, including age at arrival and duration of childhood exposure, are needed to disentangle this issue [9].

### Strengths and limitations

This study is the first large-scale community-based CE ultrasound survey in Bhutan, with a sample of 3,077 individuals across all four gewogs of Bumthang district, comparable in scale to recent surveys in Mongolia, Uzbekistan, and the Qinghai-Tibet Plateau [6, 31, 39]. We used the standardized WHO-IWGE ultrasound classification and applied two complementary approaches to account for the clustered sampling design: intercept-only logistic regression with cluster-robust standard errors (sandwich estimator) for prevalence estimation, and mixed-effects logistic regression with village-level random intercepts for risk factor analyses, in line with best practices for community-based surveys [41].

Nevertheless, this study has several limitations. The cross-sectional design precludes causal inference: CE is chronic and long-latent, and prevalent cases reflect cumulative past exposure, making it difficult to link current behaviors to infection status [42]. Key exposures were self-reported and subject to recall and social desirability bias; misclassification of complex exposures such as dog contact may have attenuated true associations toward the null [40]. The relatively small number of CE cases (*n* = 112) limited statistical power, particularly for detecting associations across multiple categorical strata, and residual confounding by unmeasured environmental exposures cannot be excluded. Abdominal ultrasound may miss very early, or heavily calcified lesions, which may lead to underestimation of the true burden [43]. In addition, abdominal ultrasound cannot assess extra-abdominal sites including the lungs. Additonally, cystic lesions (CL) - sonographically indeterminate lesions that cannot be definitively assigned to a WHO-IWGE stage were included in the overall prevalence estimate, in line with the WHO-IWGE guidance [9], as they are considered potentially echinococcal. CL lesions did not undergo confirmatory serology or follow-up imaging; validated serological assays for CE are not routinely available in Bhutan, and their diagnostic performance is further limited by documented cross-reactivity and variable sensitivity by cyst stage. Therefore, CL may not represent true CE, and their inclusion might have inflated the prevalence estimate. Finally, the questionnaire-based exposure measures may not adequately capture contact with a widely contaminated environment; integration of dog fecal testing and molecular detection of *Echinococcus *spp. eggs in soil and water would provide more objective exposure data [33]. Finally, this study was limited to one district and excluded the district hospital catchment population, where routine access to diagnostic ultrasound services is better. The prevalence estimates should therefore not be assumed to represent all districts in Bhutan. Still, because many rural communities share similar socio-cultural practices, livelihoods, and animal-contact patterns, the findings remain relevant for comparable rural settings.

## Conclusion

This community-based ultrasound survey provides the first population-level estimate of CE in Bumthang district, and demonstrates that the disease burden is substantially higher than suggested by hospital-based data. The coexistence of active and inactive cyst stages across age groups indicates ongoing transmission alongside long-standing infections, and the high proportion of previously undiagnosed cases highlights the limitations of passive surveillance. No individual-level risk factors were independently associated with prevalent CE, suggesting that transmission is primarily driven by shared environmental exposures rather than individual-level characteristics. These findings are supportive of population-wide control measures rather than targeted interventions. Effective CE control in Bhutan will require an integrated One Health approach, including regular dog deworming, vaccination of intermediate hosts, improved slaughter and offal management, strengthened environmental hygiene, community health education, and enhanced surveillance. This study establishes an important baseline for monitoring CE and provides evidence to guide integrated One Health surveillance and control strategies in Bhutan.

## Supplementary Information

Supplementary Material 1.

## Data Availability

All relevant data are within the manuscript and its Supporting Information files. The anonymized dataset is available from the corresponding author upon a reasonable request. In addition, the Ministry of Health owns the full dataset. Interested individuals may obtain the data upon request from the Chief Planning Officer, Policy and Planning Division, Ministry of Health, P.O. Box: 726, Kawangjangsa, Thimphu, Bhutan; Phone number- +975-2-328095, 321842.

## Abbreviations

aOR: Adjusted odds ratio
CE: Cystic echinococcosis
CI: Confidence interval
CL: Cystic lesion
ICC: Intraclass correlation coefficient
IQR: Interquartile range
LRT: Likelihood ratio test
NTD: Neglected tropical disease
ODK: Open Data Kit
OR: Odds ratio
PHC: Primary Health Centre
PPS: Probability proportional to size
STROBE: Strengthening the Reporting of Observational Studies in Epidemiology
WHO: World Health Organization
WHO-IWGE: WHO Informal Working Group on Echinococcosis
WHO-PEN: WHO Package of Essential Noncommunicable Disease Interventions
