# Supplementary material for: Community-based abdominal ultrasound survey of cystic echinococcosis in Bhutan: prevalence and risk factors
Source: BMC Public Health. 2026 Jun 30;26:2528. doi: 10.1186/s12889-026-28325-w (PMC13540960; doi:10.1186/s12889-026-28325-w)
Supplement: Supplementary file 1 [file 12889_2026_28325_MOESM1_ESM.docx]

**Supplementary Materials**

Table S1: Educational and Occupational Characteristics of Study Participants.

| **Education** | 2,842 |  |
| --- | --- | --- |
| No formal education |  | 1,259 (44%) |
| Secondary/High school |  | 746 (26%) |
| Primary level |  | 407 (14%) |
| Monastic education |  | 157 (5.5%) |
| Diploma/College & Above |  | 154 (5.4%) |
| Non-formal education |  | 119 (4.2%) |
| **Occupation** | 3,058 |  |
| Farmer |  | 2,139 (70%) |
| Student |  | 423 (14%) |
| Others |  | 220 (7.2%) |
| Businessperson |  | 106 (3.5%) |
| Civil servant |  | 100 (3.3%) |
| Monk/nun/monastic/military |  | 70 (2.3%) |

Table S2: Cluster-adjusted bivariate analysis for CE infection.

| Raw meat consumption | N | n CE (%) | Crude OR | 95% CI | p |
| --- | --- | --- | --- | --- | --- |
| No | 2,183 | 76 (3.36) | - | - | - |
| Yes | 566 | 16 (2.75%) | 0.84 | 0.48–1.47 | 0.50 |
| **Handwashing practice** |  |  |  |  |  |
| Yes | 2,406 | 78 (3.14%) | - | - | - |
| No | 344 | 14 (3.91%) | 1.23 | 0.68–2.22 | 0.50 |

Table S3. Individual participants questionnaire for CE ultrasound survey.

**RESPONDENT IDENTIFICATION**

Participant ID code:

Age: ……………….

Gender: ………………..

Address

- Village Code:
- Gewog Code:
- District Code:

GIS coordinates of the place:

Date of the Survey:

Name of the Interviewer: ……………………………………………..

| **No.** | **QUESTIONS** | | **RESPONSE CATEGORY** | **CODE** | **SKIP** |
| --- | --- | --- | --- | --- | --- |
|  | | **DEMOGRAPHIC AND SOCIO-ECONOMIC CHARACTERISTICS** | | | |
| 1 | What is your occupation? | | Farmer……………………………………..  Civil Servant ………………………………  Business…………………………………...  Student…………………………………….  Monk/nun…………………………………..  Military personnel…………………...........  Unemployed…………………….…………  Others (specify)…………………………... | 1  2  3  4  5  6  7  88 |  |
| 2 | What is your highest educational attainment? | | No school education……………………...  Primary/NFE……………………………….  Secondary level/High school…………….  Diploma/vocational………………….........  Monastic education……………………….  College/degree……………………………  Master’s and above………………………. | 1  2  3  4  5  6  7 |  |
| 3 | How long have you lived at the current address/place? | | Less than 1 year…………………………..  1 – 5 years…………………………………  More than 5 years………………………...  Don’t know………………………………… | 1  2  3  77 |  |
| 4 | Have you consumed alcohol within the past 12 months? | | Yes  No | 1  2 |  |
| 5 | In the past 12 months, how frequently have you had at least one drink? | | Daily…………………………………..  5-6 days a week…………………….  1-4 days a week…………………….  1-3 days a month……………………  Less than once a month…………… | 1  2  3  4  6 |  |
| 6 | Do you wash vegetables and fruits before eating? | | Always……………………………………...  Sometimes…………………………………  Never…………………….………………… | 1  2  3 |  |
| 7 | Do you wash your hands with soap prior to eating and after contacting/touching animals (dogs)? | | Always……………………………………...  Sometimes…………………………….......  Never………………………………………. | 1  2  3 |  |
| 8 | Do you eat raw or uncooked meat? | | Yes……………………………………........  No……..……………………………………  Don’t know/Not sure…………..…………. | 1  2  77 |  |
| 9 | How often do you eat raw/uncooked meat? | | Always……………………………………...  Sometimes………………………….……..  Never………………………………………. | 1  2  3 |  |
| 10 | Do you touch dogs with bare hands? | | Yes………………………………………….  No…………………………………………..  Don’t know……………………………....... | 1  2  77 |  |
| 11 | How often do you touch dogs in a month? | | Always……………………………………...  Sometimes…………………………………  Never………………………………………. | 1  2  3 |  |
| 12 | Have you heard about cystic echinococcosis before? | | Yes………………………………………….  No…………………………………………..  Don’t know…………………..……………. | 1  2  77 |  |
| 13 | Do you have a relative or family member diagnosed with CE? | | Yes………………………………………….  No…………………………………………..  Don’t know……………………………....... | 1  2  3 |  |
| 14 | Have you had any of the following symptoms in the past 30 days? | | Abdominal pain……………………………  Nausea……………………………………..  Diarrhea……………………………………  Cough………………………………………  Fever……………………………………….  Others…. (specify)………………...……..  No symptoms in past 30 days…………... | 1  2  3  4  5  88  6 |  |
| 15 | Where do you usually get information on health-related topics such as healthy lifestyle, prevention of illnesses, etc? Please tell me all your usual sources of information.   a) Health professional b) Radio (BBS, Kuzoo FM, etc) c) Television d) Internet/Social Media e) Newspaper in Dzongkha f) Newspaper in English g) Posters/leaflets h) Community meetings i) Advocacy programmes j) Peers k) Village health worker l) Others (specify) | | a) Health professional……………... b) Radio (BBS, Kuzoo FM)……….. c) Television………………………... d) Internet/Social Media………...... e) Newspaper in Dzongkha………. f) Newspaper in English………….. g) Posters/leaflets………………….. h) Community meetings…………… i) Advocacy programmes………… j) Peers………………………......... k) Village health worker…………… l) Others (specify)…………………. | 1  2  3  4  5  6  7  8  9  10  11  88 |  |

Table S4. STROBE checklist for reporting cross-sectional observational studies, completed for this manuscript.

|  | **Item No.** | **Recommendation** | **Page No.** | **Relevant text from manuscript** |
| --- | --- | --- | --- | --- |
| **Title and abstract** | **1** | (a) Indicate the study's design with a commonly used term in the title or the abstract | 1–2 | Title: "Community-based abdominal ultrasound survey of cystic Echinococcosis in Bhutan: Prevalence and risk factors"  Abstract: "We conducted a cross-sectional, community-based abdominal ultrasound survey (April–July 2025) in four gewogs of Bumthang district…" |
|  |  | (b) Provide in the abstract an informative and balanced summary of what was done and what was found | 2 | Structured abstract with Background, Methodology/Principal findings, and Conclusion/Significance sections. It reports the study design, setting, sampling approach, analytic approach (cluster-adjusted prevalence and mixed-effects regression), key prevalence estimates (overall adjusted prevalence including CL and confirmed CE), the main association (intermittent dog contact), and the programmatic implications. It also summarises incidental (non-CE) ultrasound findings and the clinical management/referral pathways for participants with CE and other clinically relevant findings. |
| **Introduction** | | | | |
| **Background/rationale** | **2** | Explain the scientific background and rationale for the investigation being reported | 4 | The Introduction section explains the global burden of CE, its neglected tropical disease status (WHO), the endemic context in Bhutan (agropastoral setting, free-roaming dogs), prior molecular evidence of E. granulosus s.l. and E. ortleppi in Bhutan, and the gap in community-level epidemiological evidence. "Epidemiological evidence on CE in Bhutan is mainly derived from hospital-based records…which provide only limited information on the true burden of disease in the community." |
| **Objectives** | **3** | State specific objectives, including any prespecified hypotheses | 4 | "This study aimed to determine the community prevalence of CE and its associated risk factors in a high-risk district in the country. By integrating epidemiological and risk factor data, this study aimed to fill existing knowledge gaps and contribute evidence toward informing national CE control strategies." |
| **Methods** | | | | |
| **Study design** | **4** | Present key elements of study design early in the paper | 5 | "This cross-sectional, community-based study was conducted between April and July 2025 in Bumthang district." The study design was clearly stated at the beginning of the Methods section under "Study Design and Setting.” |
| **Setting** | **5** | Describe the setting, locations, and relevant dates, including periods of recruitment, exposure, follow-up, and data collection | 5 | "This cross-sectional, community-based study was conducted between April and July 2025 in the Bumthang district. The Bumthang district is in the north-central region, situated at approximately 27.5°–27.9° N and 90.6°–91.1° E latitudes…Bumthang has a predominantly rural population of approximately 17,000 residents." Study sites: Four gewogs; Chamkhar town was excluded because of routine ultrasound access. |
| **Participants** | **6** | (a) Cross-sectional study—Give the eligibility criteria, and the sources and methods of selection of participants | 6 | Eligibility: "Eligible participants were individuals aged ≥12 years who had resided in the selected households for at least six months, provided informed consent (or assent for minors), and were present during the survey. Individuals with acute or severe illnesses…and temporary residents were excluded."  Sampling: Three-stage cluster design using probability proportional to size (PPS): Stage 1 — 15/20 chiwogs selected by PPS; Stage 2 — approximately three villages per chiwog (44 total); Stage 3 — all eligible household members invited. |
|  |  | (b) Cohort/Case-control—For matched studies, give matching criteria and number of exposed and unexposed / controls per case | N/A | Not applicable (cross-sectional study; no matching was performed). |
| **Variables** | **7** | All outcomes, exposures, predictors, potential confounders, and effect modifiers were clearly defined. Give diagnostic criteria, if applicable | 7 | Outcomes: "Confirmed CE cases were defined as 'individuals with ultrasound findings pathognomonic of echinococcal cysts'; suspected cases were defined as 'ultrasound findings consistent with, but not pathognomonic of CE cysts' (including CL lesions). Both combined into a single binary outcome (CE vs. non-CE)."  The exposures/predictors were sex, age group (<19, 19–39, 40–59, and ≥60 years), education, occupation, place of birth, dog contact frequency, raw meat consumption, and handwashing. The WHO-IWGE classification was used for cyst staging. |
| **Data sources/ measurement** | **8*** | For each variable of interest, provide the sources of data and details of the assessment methods (measurement). Describe comparability of assessment methods if there is more than one group | 5–7 | CE outcome: Abdominal ultrasound using Mindray M6 and MX7 machines with curvilinear transducers, a standardized B-mode scanning protocol, and the WHO-IWGE classification. The procedure was performed by the PI and an experienced sonographer (>10 years). Subset double-read for inter-observer agreement.  Exposure: A structured questionnaire on sociodemographic characteristics, dog-related practices, food handling/hygiene behaviors, environmental exposures, and CE awareness. This was completed before the ultrasound. Collected via ODK Collect, with validation rules applied. |
| **Bias** | **9** | Describe any efforts to address potential sources of bias | 7, 18–19 | Efforts to minimize bias: trained sonographers and data collectors, pre-session briefings, standardized scanning protocol, daily completeness checks by the PI, independent double-reading of CE cyst images for inter-observer agreement, and data validation rules in ODK.  Residual bias discussed in Limitations: self-reported exposures subject to recall and social desirability bias; misclassification of dog contact may have attenuated associations; cross-sectional design means exposures reflect current, not historical behavior. |
| **Study size** | **10** | Explain how the study size was arrived at | 6 | "The sample size was calculated to estimate the CE prevalence with a precision of two percentage points (half-width of the 95% CI), assuming a prevalence of 4% and an intraclass correlation coefficient of 0.05. With an average cluster size of approximately 30 individuals, the corresponding design effect was 2.45 [DEFF = 1 + (30−1) × 0.05], requiring a minimum of 38 clusters and 1,140 individuals; inflated by 10% to account for non-response, the final minimum sample size was 1,254." |
| **Quantitative variables** | **11** | Please explain how quantitative variables were handled in the analyses. If applicable, describe which groupings were chosen and why | 7 | Age was categorised as <19, 20–39, 40–59, and ≥60 years. Education, occupation, place of birth, and dog-contact frequency were analysed as categorical variables as collected in the questionnaire (with minor harmonisation for analysis as described in the Methods). Cyst size (largest cyst diameter) was described at the person level and categorised as <5 cm, 5–9.9 cm, and ≥10 cm. |
| **Statistical methods** | **12** | (a) Describe all statistical methods, including those used to control for confounding | 7–8 | Prevalence: Intercept-only logistic regression with cluster-robust standard errors (sandwich estimator at the village level), back-transformed using inverse logit. For zero-count subgroups: exact binomial (Clopper–Pearson) CIs.  Bivariate analysis: Mixed-effects logistic regression with village-level random intercept; crude odds ratios (ORs) and 95% Wald confidence intervals (CIs). LRT for global p-values (cutoff p<0.25 for candidate selection).  Multivariable: Mixed-effects logistic regression with village-level random intercept (lme4, BOBYQA optimizer, 200,000 evaluations). Age and sex were retained a priori. ICC calculated as σ²ᵤ/(σ²ᵤ + π²/3). |
|  |  | (b) Describe any methods used to examine subgroups and interactions | 7–8 | Age- and sex-specific prevalence estimates were derived using subgroup-specific intercept-only models with cluster-robust standard errors. Geographic (gewog-level) prevalence was also computed. No formal interaction terms were tested, and subgroup analyses were reported descriptively. |
|  |  | (c) Explain how missing data were addressed | 7–8 | "Bivariate and risk factor analyses were restricted to participants with complete covariate data (n = 3,058)." Of the 3,077 screened, 19 were excluded due to missing village identifiers or covariate data. A complete case analysis was performed. |
|  |  | (d) Cross-sectional study—If applicable, describe analytical methods taking account of sampling strategy | 7–8 | Clustered sampling design explicitly accounted for using: (1) intercept-only logistic regression with cluster-robust standard errors (sandwich estimator) for prevalence estimation; and (2) mixed-effects logistic regression with village-level random intercepts for bivariate and multivariable analyses. The design effect and ICC were incorporated into the analysis framework. |
|  |  | (e) Describe any sensitivity analyses | 8 | "As a sensitivity analysis, alternative clustering structures were explored, including household-level clustering and households nested within the villages. These models were compared using the Akaike Information Criterion (AIC), Bayesian Information Criterion (BIC), and likelihood ratio tests. The village-only random intercept model was retained as the primary analysis model as household variance was statistically unstable due to sparse binary data, a median household size of two participants and a rare outcome (~3.6% prevalence)." |
| **Results** | | | | |
| **Participants** | **13*** | (a) Report numbers of individuals at each stage of study—eg numbers potentially eligible, examined for eligibility, confirmed eligible, included in the study, completing follow-up, and analysed | 9–10 | Total screened: 3,077 individuals from 44 villages across four gewogs. Included in the analyses: 3,058 (participants with complete covariate data and village identifiers). Number of CE cases identified: 112 (confirmed or suspected). "Among the 3,077 screened participants, 112 were identified to have CE." A total of 1,266 distinct households were included in the study. |
|  |  | (b) Give reasons for non-participation at each stage | 6, 9 | All eligible individuals in selected households were invited; 3,077 individuals were screened. Nineteen participants were excluded from the analysis due to incomplete data (missing village identifier and/or covariates required for analysis). Reasons for non-participation among invited individuals (if any) were not captured in a structured manner beyond the eligibility exclusions described in the Methods. |
|  |  | (c) Consider use of a flow diagram | 9 | Figure 1 shows a map of the study site, including the district hospital, PHCs, and community centers for participant recruitment. A formal CONSORT-style participant flow diagram is not included; however, the text describes the participants at key stages (eligible criteria, n screened, n analyzed, n with CE). |
| **Descriptive data** | **14*** | (a) Give characteristics of study participants (eg demographic, clinical, social) and information on exposures and potential confounders | 9–10, 12–13 | Table 1: Baseline characteristics — sex (60% female), age (median 43 years; 56% aged ≥40), place of origin (82% native of Bumthang), household size (median 2, IQR 1–18). Table 2: Distribution of exposures and potential confounders (education, occupation, place of birth, dog contact) by CE status. |
|  |  | (b) Indicate number of participants with missing data for each variable of interest | 9 | Missingness is reported at the analysis level: of 3,077 screened participants, 3,058 had complete data for prevalence/risk-factor analyses (19 excluded). For multivariable modelling, complete-case data were used (n = 2,842), as noted in the Results/Table 2 legend (“Numbers (N) vary by variable due to missing data; multivariable models were fitted using complete-case data (n = 2,842)”). Per-variable missing counts are not tabulated. |
|  |  | (c) Cohort study—Summarise follow-up time (eg, average and total amount) | N/A | Not applicable (cross-sectional study; no follow-up). |
| **Outcome data** | **15*** | Cross-sectional study—Report numbers of outcome events or summary measures | 10 | "Among the 3,077 screened individuals, 112 were identified as having CE (confirmed or suspected). After adjusting for village-level clustering, the overall adjusted prevalence of CE was 3.66% (95% CI: 2.92–4.59); the cluster-adjusted prevalence of confirmed CE was 2.58% (95% CI: 1.93–3.44), while the cluster-adjusted prevalence of suspected CE was 1.08% (95% CI: 0.76–1.53). Of the 79 confirmed CE cases, 26.6% (21/79) had been previously diagnosed." |
| **Main results** | **16** | (a) Provide unadjusted estimates and, if applicable, confounder-adjusted estimates and their precision (e.g., 95% confidence interval). Make clear which confounders were adjusted for and why they were included | 13–15 | Table 2 reports crude ORs (from mixed-effects logistic regression with a village-level random intercept) and adjusted ORs (aORs) with 95% CIs from a multivariable mixed-effects logistic regression model with a village-level random intercept. Sex and age were retained a priori; additional covariates included in the final model were selected using a likelihood ratio test screening criterion (p<0.25) and are reported in Table 2 (education, occupation, place of birth, and dog-contact frequency). |
|  |  | (b) Report category boundaries when continuous variables were categorized | 7, 13 | Age: <19, 20–39, 40–59, ≥60 years. Cyst size: <5 cm, 5–9.9 cm, ≥10 cm. Dog contact frequency: never, rarely, sometimes, often, always. Education categories are presented as collected (no formal, non-formal, primary, secondary/high school, monastic, diploma/college & above). Category boundaries are described in the Methods and reflected in the Results tables/figures. |
|  |  | (c) If relevant, consider translating estimates of relative risk into absolute risk for a meaningful time period | 10 | Prevalence estimates are provided as absolute risk (proportion) — overall adjusted prevalence 3.66%, confirmed CE 2.58%) rather than relative risk. The absolute prevalence by age, sex, and gewog was also reported. Not translated into incidence rates (cross-sectional study). |
| **Other analyses** | **17** | Report other analyses done—eg analyses of subgroups and interactions, and sensitivity analyses | 8, 10–11, 18 | Subgroup analyses: Age- and sex-specific cluster-adjusted prevalence estimates (Figure 2). Gewog-level prevalence estimates (Figure 3, ranging from 1.39% to 4.92%). Dog ownership subgroup prevalence was estimated.  Sensitivity analyses: Alternative clustering structures (household, household-within-village) compared to the village-only model via AIC, BIC, and LRT; village-only model retained.  Discussion of negative confounding by age, education, and occupation for the dog contact variable. |
| **Discussion** | | | | |
| **Key results** | **18** | Summarise key results with reference to study objectives | 16 | "This community-based ultrasound survey, the first of its kind in Bhutan, screened 3,077 individuals aged >12 years across 44 villages in Bumthang district. The overall cluster-adjusted CE prevalence was 3.66% (95% CI: 2.92–4.59)…confirms that CE is a substantial yet largely under-recognized public health problem in Bhutan." Key findings on cyst characteristics, risk factors (intermittent dog contact aOR 1.73), and village-level clustering (ICC ~3.3%) were summarized in relation to the objectives. |
| **Limitations** | **19** | The limitations of the study are discussed, taking into account the sources of potential bias or imprecision. Discuss both direction and magnitude of any potential bias | 18–19 | "This study had several limitations. The cross-sectional design precludes causal inference…Key exposures were self-reported and subject to recall and social desirability bias; misclassification of complex exposures, such as dog contact, may have attenuated true associations toward the null…The relatively small number of CE cases (n = 112) limited the statistical power (wide confidence intervals)…Abdominal ultrasound…may miss very early, heavily calcified, or extra-abdominal disease, slightly underestimating the true burden…CL may not represent true CE, and their inclusion…may overestimate the true CE burden." |
| **Interpretation** | **20** | Give a cautious overall interpretation of results considering objectives, limitations, multiplicity of analyses, results from similar studies, and other relevant evidence | 16–20 | The discussion provides a cautious interpretation of the findings in the context of comparable studies from Tibet, Mongolia, the Qinghai-Tibet Plateau, and Kyrgyzstan. Negative confounding of dog contact associations was explained. The ICC of 3.3% was interpreted in the context of focal endemicity. Pattern of weak individual-level risk factors framed as "environmentally-mediated widespread risk." The findings support One Health integrated strategies rather than targeted individual-level interventions. |
| **Generalisability** | **21** | Discuss the generalisability (external validity) of the study results | 19–20 | "While these findings provide a solid baseline for the Bumthang district, extrapolation to other parts of Bhutan should be approached with caution, given the country's ecological and socio-cultural diversity. Expanding community-based ultrasound surveillance to other endemic districts would enable a more comprehensive national picture and support geographically targeted control strategies." |
| **Other information** | | | | |
| **Funding** | **22** | Give the source of funding and the role of the funders for the present study and, if applicable, for the original study on which the present article is based | 22 | Funding is reported in the Declarations section: “Rudolf Geigy Foundation (R. Geigy Stiftung: Die Stiftung des Swiss TPH), Switzerland (Grant award – Basel, 11. Dezember 2024).” The manuscript does not specify the role of the funder(s) in study design, data collection, analysis, interpretation, or manuscript preparation. |
